# Sensitivity to orientation is not unique to social attention cueing

**DOI:** 10.1038/s41598-022-09011-4

**Published:** 2022-03-23

**Authors:** Tim Vestner, Katie L. H. Gray, Richard Cook

**Affiliations:** 1 Department of Psychological Sciences, Birkbeck, University of London, Malet Street, London, WC1E7HX UK; 2 School of Psychology and Clinical Language Sciences, University of Reading, Reading, UK; 3 Department of Psychology, University of York, York, UK

**Keywords:** Psychology, Human behaviour

## Abstract

It is well-established that faces and bodies cue observers’ visuospatial attention; for example, target items are found faster when their location is cued by the directionality of a task-irrelevant face or body. Previous results suggest that these cueing effects are greatly reduced when the orientation of the task-irrelevant stimulus is inverted. It remains unclear, however, whether sensitivity to orientation is a unique hallmark of “social” attention cueing or a more general phenomenon. In the present study, we sought to determine whether the cueing effects produced by common objects (power drills, desk lamps, desk fans, cameras, bicycles, and cars) are also attenuated by inversion. When cueing stimuli were shown upright, all six object classes produced highly significant cueing effects. When shown upside-down, however, the results were mixed. Some of the cueing effects (e.g., those induced by bicycles and cameras) behaved liked faces and bodies: they were greatly reduced by orientation inversion. However, other cueing effects (e.g., those induced by cars and power drills) were insensitive to orientation: upright and inverted exemplars produced significant cueing effects of comparable strength. We speculate that (i) cueing effects depend on the rapid identification of stimulus directionality, and (ii) some cueing effects are sensitive to orientation because upright exemplars of those categories afford faster processing of directionality, than inverted exemplars. Contrary to the view that attenuation-by-inversion is a unique hallmark of social attention, our findings indicate that some non-social cueing effects also exhibit sensitivity to orientation.

## Introduction

When viewing human faces and bodies in visual scenes, the directionality of the stimuli cue participants’ visuospatial attention^1–3^. For example, participants find target items faster when the correct location (on the left or right of the display) is cued by the directionality (facing-left vs. facing-right) of a task-irrelevant face^4^ or body^5^. A tendency to orient our attention to parts of the environment attended by others may help us discover items of interest or value, and identify potential threats^6^. Interestingly, however, several findings suggest that these cueing effects are sensitive to the orientation of the cueing stimulus; when faces and bodies are shown upside-down, the cueing effects induced are reduced or abolished^4,5,7–9^.

To date, it remains unclear why stimulus inversion disrupts social attention cueing. One possible explanation of the inversion effects observed is that domain-specific mechanisms for gaze following are tuned to upright faces and bodies, and are no-longer engaged—or engaged less—by inverted exemplars. According to one domain-specific account, humans possess innate neurocognitive mechanisms for gaze following^10–12^. It is likely that following the gaze of others helps to scaffold the development of more complex mentalizing skills (e.g., visual perspective taking, understanding of others’ beliefs and desires), thought to aid social learning, interaction and collaboration^13^. A tendency to follow the gaze of others may have therefore conveyed on our ancestors a competitive advantage. As such, the neurocognitive mechanisms responsible may have become genetic adaptations. These mechanisms may be tuned to the upright orientation of faces and bodies because this is how our ancestors encountered conspecifics. Somewhat consistent with this view, newborn infants just a few hours old will orient toward face-like patterns shown upright, but not upside-down^14,15^.

The processing delay hypothesis articulates a different view: inverted faces may not cue attention as effectively as upright faces, because inverted faces are processed less efficiently^5^. Faces and bodies are usually encountered in their canonical orientation. The visual processing of upright exemplars may therefore benefit from perceptual expertise, in a way that the visual processing of inverted exemplars does not^16–18^. According to the processing delay hypothesis, cueing effects depend on the rapid processing and identification of stimulus directionality. Inverted faces and bodies are thought to be ineffective attention cues because the processing of inverted exemplars is slower than the processing of upright exemplars. Consistent with this view, findings from EEG paradigms indicate that orientation inversion delays the visual processing of faces^19^ and bodies^20^. Insofar as the perceptual processing of non-social stimuli can also be delayed by inversion^21^, the processing delay account is domain-general.

The present study sought to better understand the orientation specificity of attention cueing. In our first set of experiments, we confirmed that faces and bodies cue attention when shown upright but not when shown upside-down. Next, we examined whether cueing effects produced by common objects (power drills, desk lamps, desk fans, cameras, bicycles, and cars) also show orientation-specificity. Evidence that orientation-specificity is a unique feature of the cueing effects produced by faces and bodies would accord with the view that domain-specific mechanisms for gaze-following are tuned to upright exemplars. Conversely, evidence that non-social cueing effects also show orientation-specificity would suggest that orientation-specificity is a more general phenomenon.

## Cueing of visuospatial attention by faces and bodies

To begin our investigation, we conducted three experiments to confirm previous reports that the attention cueing effects produced by faces and bodies are greatly reduced by orientation inversion^4,5^. One of our experiments employed face stimuli. The two other experiments employed body stimuli. In one experiment, we used whole bodies with the head and face visible. In another, we used body images with the head and face occluded. This was to determine whether any effects of orientation on body cueing were independent of head/face inversion^22^.

All the experiments described were conducted online, an approach that is increasingly common. Evidence suggests that carefully-designed online tests of cognitive and perceptual processing can yield high-quality data, comparable with that collected in the lab^23–25^. All experiments used the same cueing procedure (described in detail below). We elected to use this task having previously established that it produces clear, consistent effects in online studies^5,26^. This was also the procedure that first revealed attention cueing effects by common objects^26^.

For each experiment, cue validity (valid, invalid) was manipulated within-subjects, while stimulus orientation (upright, inverted) was manipulated between-subjects. Relative to within-subjects designs, between-subjects designs necessitate larger sample sizes to achieve comparable statistical power. However, manipulating orientation between-subjects let us establish beyond any doubt whether upright and inverted exemplars cue attention. It is conceivable that inverted exemplars do not spontaneously cue the attention of participants, but can acquire some cueing ability by being interleaved with upright exemplars. It is also possible that interleaving upright and inverted exemplars suppresses the cueing effects seen for inverted exemplars (e.g., participants may anticipate that their performance is expected to differ in the two conditions). By using a between-subjects approach, however, we can be confident that our results are unaffected by any carry-over effects.

### Methods

#### Participants

For all of the experiments described, the participants were recruited through Prolific (www.prolific.co). To be eligible to participate, individuals had to be aged 18 to 60 years-old, speak fluent English, have normal or corrected-to-normal visual acuity, and have no history of psychiatric or neurological illness (e.g., autism spectrum disorder, schizophrenia). All participants had a Prolific approval rate of at least 75%. The inclusion criteria were identified at the outset and were the same for all experiments. All of the experiments described were completed by separate groups of participants; i.e., each sample was completely independent. No-one was replaced or excluded in any of the experiments described.

The sample size for each experiment (N = 120) was determined a priori using a power analysis. We assumed an effect size of f = 0.3 and a target power of 0.95. This analysis yielded a target sample size of 112, which was rounded up to 120. Ethical clearance was granted by the local ethics committee (the Departmental Ethics Committee for Psychological Sciences, Birkbeck, University of London) and the experiment was conducted in line with the ethical guidelines laid down in the 6th (2008) Declaration of Helsinki. All participants gave informed consent. None of the experiments described were preregistered.

#### Stimuli

The images used in the face experiment were sourced from the Radboud Face Database^27^. The images used in the two body experiments were sourced from the Adobe Stock Service. Eight different exemplars were used in each experiment. We created mirror images of each exemplar so that it could be presented facing left or right. Images were standardized to a height of 400 pixels. The faces and bodies were approximately ~ 350 pixels and ~ 175 pixels wide, respectively. Adobe Photoshop was used to place a grey oval over the head and face of the body images to produce the occluded stimuli. The stimulus images used in each of the experiments described can be accessed at https://osf.io/sx7tv/.

The stimulus images used in the face and body (head occluded) experiments were identical to those used by Vestner, Gray, and colleagues^5^, while the images used in the body (head visible) experiment were those employed by Vestner, Over, and colleagues^26^. The stimuli used in the face experiment also closely resemble those employed by Langton and Bruce^4^.

#### Procedure

Experimental trials began with a fixation cross in the center of the screen. After 2000 ms, a cueing stimulus appeared in the center, replacing the fixation cross. On 50% of trials, the cueing stimulus faced rightwards, on 50% of trials, the cueing stimulus faced leftwards. For half the participants (N = 60), this cueing stimulus appeared upright. For the other half (N = 60), this cueing stimulus appeared upside-down. After a further 500 ms, two letter arrays appeared on screen, one on the left and one on the right, each consisting of 6 letters arranged vertically. Participants were shown a target letter at the beginning of each block of trials, which was chosen randomly from a pool of 13 letters [E, F, H, K, L, M, N, T, V, W, X, Y, Z] selected for their linear components and angular features. The remaining letters were used to populate the arrays.

**Figure 1:**
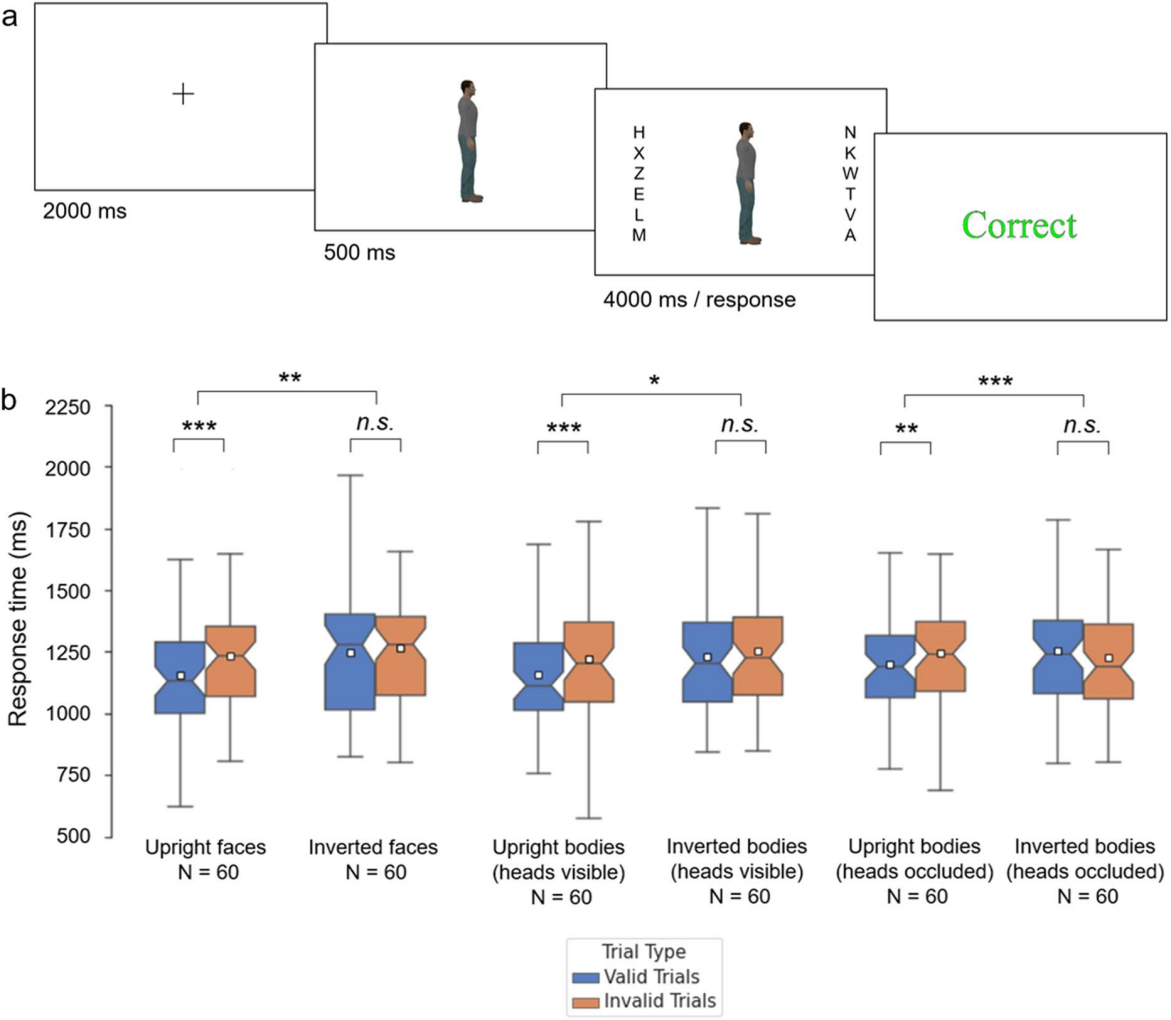
(**a**) Structure of a trial from the bodies (heads visible) experiment. The body image included in this schematic was not used as a stimulus image but is representative of the stimuli employed. This image was created by the authors for illustrative purposes. (**b**) Results from the cueing experiments conducted with faces and bodies. Boxes indicate inter-quartile range. Notches indicate confidence interval of the median. Whiskers indicate 1.5 * interquartile range. White squares denote the mean. *** denotes significance at *p* < .001. ** denotes significance at *p* < .025. * denotes significance at *p* < .05.

Participants were asked to press spacebar if the target letter was on screen. On catch trials, the target letter was not presented and participants were instructed to make no response. In either case, trials ended after 4000 ms. After response or time-out, participants were given feedback: either “Correct”, “Incorrect”, or “Too slow”. In total, the procedure consisted of eight blocks of 24 trials. See Fig. 1a for an overview of the procedure. Each block comprised 8 valid trials (the central stimulus cued the array containing the target letter), 8 invalid trials (the central stimulus cued the array that did not contain the target letter), and 8 catch trials (the target letter was not present).

The experimental tasks were programmed in Unity3D (Version 2018.3.7f1) and were compiled using WebGL. The experiments were hosted on an Amazon Lightsail server. Response times (RTs) were recorded locally on participants’ computers and therefore not influenced by differences in data transmission speeds. This method is known to produce similar RT distributions to those seen in the laboratory^28^. The procedure used, including the stimulus offset asynchrony (SOA) of 500 ms, has been shown to produce clear and reliable cueing effects for faces^5,26^, bodies^5,26^, and objects^26^ when administered online.

### Results

The results from this series of experiments are depicted in Fig. 1b. The underlying data can be accessed at https://osf.io/sx7tv/. For each type of stimulus, a directional cueing effect is inferred from faster RTs on valid trials than on invalid trials. This was assessed through a paired-samples *t*-test (α = 0.05, two-tailed).

#### Faces

120 participants (58 female, 62 male) with an age range of 18 to 59 years (*M*_age_ = 29.0, *SD*_age_ = 9.7) were recruited through Prolific. Trials where participants responded incorrectly (1.5%) were excluded from the analysis. All participants performed correctly on at least 60 of the 64 catch trials. RTs were subjected to ANOVA with Validity (valid, invalid) as a within-subjects factor and Orientation (upright, inverted) as a between-subjects factor. The analysis revealed a significant main effect of Validity [*F*(1,118) = 15.38, *p* < 0.001, η_p_^2^ = 0.115], whereby participants responded faster on valid trials, than on invalid trials. We observed no main effect of Orientation [*F*(1,118) = 0.66, *p* = 0.419, η_p_^2^ = 0.006]. There was a significant Validity × Orientation interaction [*F*(1,118) = 5.46, *p* = 0.021, η_p_^2^ = 0.044]. Further comparisons revealed a significant cueing effect for upright faces [*t*(59) = 4.16, *p* < 0.001, *d* = 0.541, CI_95%_ = 0.04, 0.12], but not for inverted faces [*t*(59) = 1.20, *p* = 0.233, *d* = 0.156, CI_95%_ = − 0.01, 0.05].

#### Bodies (heads visible)

120 participants (66 female, 53 male, 1 non-binary) with an age range of 18 to 60 years (*M*_age_ = 30.7, *SD*_age_ = 10.4) were recruited through Prolific. Trials where participants responded incorrectly (1.7%) were excluded from the analysis. All participants performed correctly on at least 60 of the 64 catch trials. RTs were subjected to ANOVA with Validity (valid, invalid) as a within-subjects factor and Orientation (upright, inverted) as a between-subjects factor. The analysis revealed a significant main effect of Validity [*F*(1,118) = 18.19, *p* < 0.001, η_p_^2^ = 0.134], whereby participants responded faster on valid trials, than on invalid trials. We observed no main effect of Orientation [*F*(1,118) = 1.72, *p* = 0.193, η_p_^2^ = 0.014]. There was a significant Validity × Orientation interaction [*F*(1, 118) = 4.63, *p* = 0.033, η_p_^2^ = 0.038]. Further comparisons revealed a significant cueing effect for upright bodies [*t*(59) = 4.63, *p* < 0.001, *d* = 0.600, CI_95%_ = 0.04, 0.09], but not for inverted bodies [*t*(59) = 1.47, *p* = 0.148, *d* = 0.193, CI_95%_ = − 0.01, 0.05].

#### Bodies (heads occluded)

120 participants (51 female, 66 male, 3 non-binary) with an age range of 18 to 60 years (*M*_age_ = 31.5, *SD*_age_ = 10.3) were recruited through Prolific. Trials where participants responded incorrectly (1.5%) were excluded from the analysis. All participants performed correctly on at least 60 of the 64 catch trials. RTs were subjected to ANOVA with Validity (valid, invalid) as a within-subjects factor and Orientation (upright, inverted) as a between-subjects factor. The analysis revealed no main effect of Validity [*F*(1,118) = 0.95, *p* = 0.332, η_p_^2^ = 0.008] and no main effect of Orientation [*F*(1,118) = 0.21, *p* = 0.647, η_p_^2^ = 0.002]. There was a significant Validity × Orientation interaction [*F*(1, 118) = 12.87, *p* < 0.001, η_p_^2^ = 0.098]. Further comparisons revealed a significant cueing effect for upright bodies [*t*(59) = 3.14, *p* = 0.003, *d* = 0.404, CI_95%_ = 0.02, 0.08], but not for inverted bodies [*t*(59) = 1.90, *p* = 0.062, *d* = 0.250, CI_95%_ = − 0.001, 0.05].

## Cueing of visuospatial attention by common objects

The results from our first three experiments were remarkably consistent. As expected, we observed significant Validity × Orientation interactions in all three experiments. Participants responded faster on valid trials than on invalid trials when the cueing stimulus was upright (indicative of attention cueing), but not when the cueing stimulus was upside-down.

Next, we sought to determine if this pattern is a unique hallmark of social attention cueing. Until recently, it was not possible to interrogate this view. Only a few non-social stimuli—including arrows^29,30^ and hand tools^31^—were known to cue observers’ attention, and these cueing stimuli do not possess a canonical orientation. For example, arrows are typically symmetric about their horizontal axis. Similarly, tools such as screwdrivers and hammers are frequently encountered in a range of different orientations. In the absence of a ‘right way up’ it is impossible to present something ‘upside-down’.

**Figure 2:**
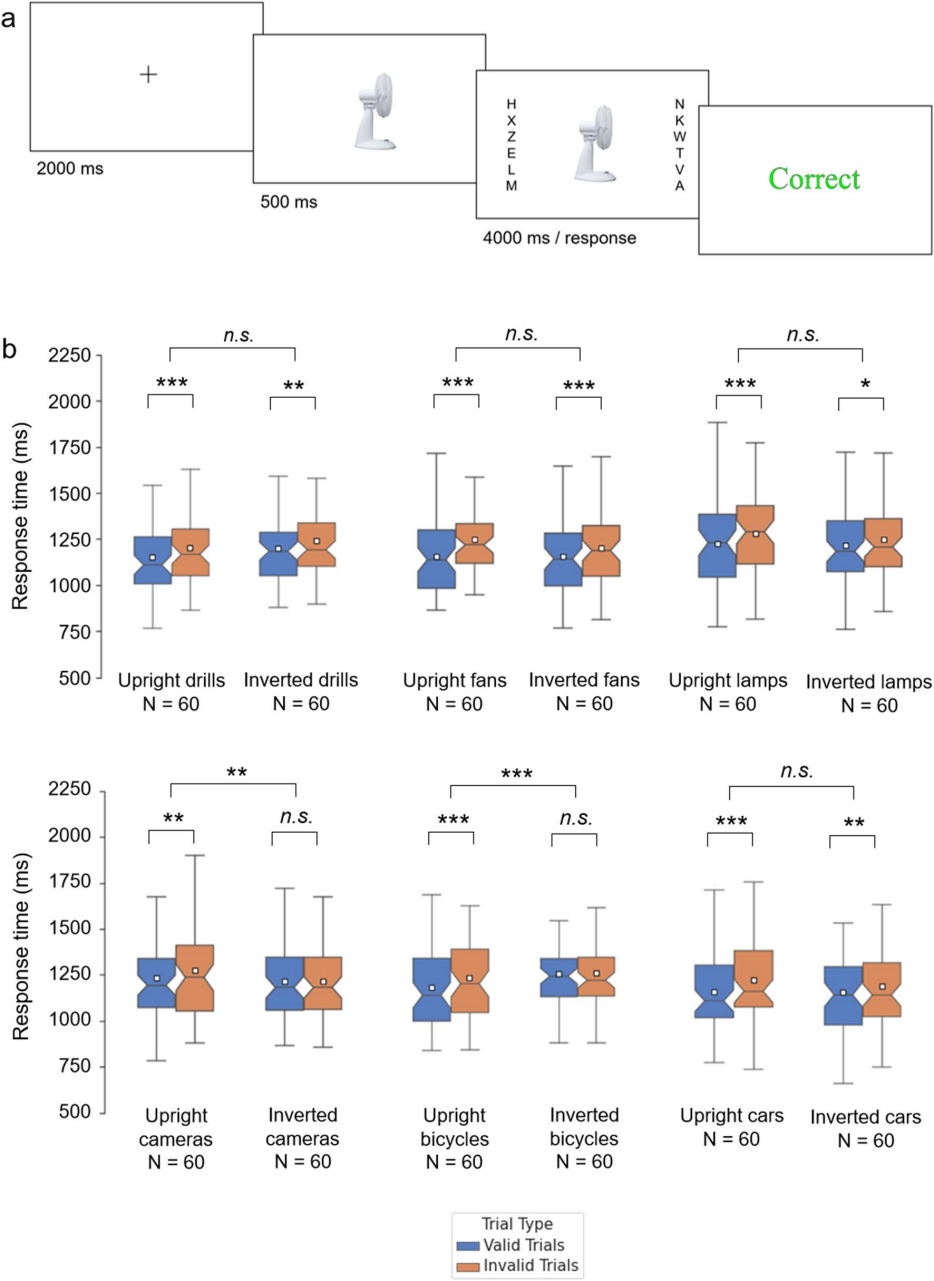
(**a**) Structure of a trial from the desk fans experiment. The fan image included in this schematic was not used as a stimulus image but is representative of the stimuli employed. This image was created by the authors for illustrative purposes. (**b**) Results from the cueing experiments conducted with common objects. Boxes indicate inter-quartile range. Notches indicate confidence interval of the median. Whiskers indicate 1.5 * interquartile range. White squares denote the mean. *** denotes significance at *p* < .001. ** denotes significance at *p* < .025. * denotes significance at *p* < .05.

However, a recent paper by Vestner, Over, and colleagues^26^ described attention cueing effects for a range of common objects, namely power drills, desk fans, desk lamps, cameras, bicycles, and cars, when viewed in profile. Importantly, each of these objects has a right way up. We therefore conducted six further experiments to determine whether the cueing effects induced by these objects also exhibit orientation sensitivity. With the exception of the cueing stimuli used (objects rather than faces and bodies), the method and procedure were identical to our first three experiments (Fig. 2a).

### Methods

The stimulus images used in each of the six experiments were sourced from various websites. Eight different exemplars were used in each experiment. We created mirror images of each exemplar so that it could be presented facing left or right. Images were standardized to a height of 400 pixels (power drills, desk fans, desk lamps, cameras) or 200 pixels (bicycles, cars). The drills, desks fans, desk lamps, and cameras were approximately ~ 350 pixels, ~ 270 pixels, ~ 200 pixels, and ~ 200 pixels wide, respectively. The bicycles and cars were approximately ~ 625 pixels and ~ 530 pixels wide, respectively. The images used in these six experiments are identical to those employed by Vestner, Over, and colleagues^26^.

### Results

The results from this series of experiments are depicted in Fig. 2b. The underlying data can be accessed at https://osf.io/sx7tv/. For each type of stimulus, a directional cueing effect is inferred from faster RTs on valid trials than on invalid trials. This was assessed through a paired-samples *t*-test (α = 0.05, two-tailed).

#### Power drills

120 participants (60 female, 59 male, 1 non-binary) with an age range of 18 to 60 years (*M*_age_ = 33.4, *SD*_age_ = 10.9) were recruited through Prolific. Trials where participants responded incorrectly (1.5%) were excluded from the analysis. All participants performed correctly on at least 60 of the 64 catch trials. RTs were subjected to ANOVA with Validity (valid, invalid) as a within-subjects factor and Orientation (upright, inverted) as a between-subjects factor. The analysis revealed a significant main effect of Validity [*F*(1,118) = 16.38, *p* < 0.001, η_p_^2^ = 0.122], whereby participants responded faster on valid trials, than on invalid trials. We observed no main effect of Orientation [*F*(1,118) = 0.07, *p* = 0.795, η_p_^2^ = 0.001] and no Validity × Orientation interaction [*F*(1, 118) = 0.44, *p* = 0.509, η_p_^2^ = 0.004]. Further comparisons revealed significant cueing effects for upright [*t*(59) = 2.84, *p* < 0.001, *d* = 0.369, CI_95%_ = 0.02, 0.09] and inverted power drills [*t*(59) = 3.03, *p* = 0.004, *d* = 0.389, CI_95%_ = 0.01, 0.06].

#### Desk fans

120 participants (61 female, 56 male, 3 non-binary) with an age range of 18 to 59 years (*M*_age_ = 30.5, *SD*_age_ = 11.1) were recruited through Prolific. Trials where participants responded incorrectly (1.6%) were excluded from the analysis. All participants performed correctly on at least 60 of the 64 catch trials. RTs were subjected to ANOVA with Validity (valid, invalid) as a within-subjects factor and Orientation (upright, inverted) as a between-subjects factor. The analysis revealed a significant main effect of Validity [*F*(1,118) = 40.88, *p* < 0.001, η_p_^2^ = 0.257], whereby participants responded faster on valid trials, than on invalid trials. We observed no main effect of Orientation [*F*(1,118) = 0.55, *p* = 0.462, η_p_^2^ = 0.005] and no Validity × Orientation interaction [*F*(1,118) = 3.72, *p* = 0.056, η_p_^2^ = 0.031]. Further comparisons revealed significant cueing effects for upright [*t*(59) = 5.26, *p* < 0.001, *d* = 0.679, CI_95%_ = 0.06, 0.12] and inverted desk fans [*t*(59) = 3.65, *p* = 0.001, *d* = 0.475, CI_95%_ = 0.02, 0.07].

#### Desk lamps

120 participants (51 female, 68 male, 1 non-binary) with an age range of 18 to 59 years (*M*_age_ = 31.9, *SD*_age_ = 11.2) were recruited through Prolific. Trials where participants responded incorrectly (1.4%) were excluded from the analysis. All participants performed correctly on at least 60 of the 64 catch trials. RTs were subjected to ANOVA with Validity (valid, invalid) as a within-subjects factor and Orientation (upright, inverted) as a between-subjects factor. The analysis revealed a significant main effect of Validity [*F*(1,118) = 15.56, *p* < 0.001, η_p_^2^ = 0.117], whereby participants responded faster on valid trials, than on invalid trials. We observed no main effect of Orientation [*F*(1,118) = 0.59, *p* = 0.443, η_p_^2^ = 0.005] and no Validity × Orientation interaction [*F*(1,118) = 1.37, *p* = 0.245, η_p_^2^ = 0.011]. Further comparisons revealed significant cueing effects for upright [*t*(59) = 3.34, *p* = 0.001, *d* = 0.432, CI_95%_ = 0.02, 0.09] and inverted desk lamps [*t*(59) = 2.16, *p* = 0.035, *d* = 0.282, CI_95%_ = 0.002, 0.06].

#### Cameras

120 participants (74 female, 46 male) with an age range of 18 to 60 years (*M*_age_ = 30.6, *SD*_age_ = 10.9) were recruited through Prolific. Trials where participants responded incorrectly (1.9%) were excluded from the analysis. All participants performed correctly on at least 60 of the 64 catch trials. RTs were subjected to ANOVA with Validity (valid, invalid) as a within-subjects factor and Orientation (upright, inverted) as a between-subjects factor. The analysis revealed a significant main effect of Validity [*F*(1,118) = 4.73, *p* = 0.032, η_p_^2^ = 0.039], whereby participants responded faster on valid trials, than on invalid trials. We observed no main effect of Orientation [*F*(1,118) = 1.22, *p* = 0.272, η_p_^2^ = 0.010]. However, there was a significant Validity × Orientation interaction [*F*(1,118) = 6.51, *p* = 0.012, η_p_^2^ = 0.052]. Further comparisons revealed significant cueing effects for upright cameras [*t*(59) = 3.29, *p* = 0.002, *d* = 0.425, CI_95%_ = 0.01, 0.06], but not for inverted cameras [*t*(59) = 0.27, *p* = 0.787, *d* = 0.036, CI_95%_ = − 0.02, 0.02].

#### Bicycles

120 participants (65 female, 53 male, 2 non-binary) with an age range of 18 to 57 years (*M*_age_ = 30.4, *SD*_age_ = 10.7) were recruited through Prolific. Trials where participants responded incorrectly (1.9%) were excluded from the analysis. All participants performed correctly on at least 59 of the 64 catch trials. RTs were subjected to ANOVA with Validity (valid, invalid) as a within-subjects factor and Orientation (upright, inverted) as a between-subjects factor. The analysis revealed a significant main effect of Validity [*F*(1,118) = 17.2, *p* < 0.001, η_p_^2^ = 0.127], whereby participants responded faster on valid trials, than on invalid trials. We observed no main effect of Orientation [*F*(1,118) = 0.48, *p* = 0.490, η_p_^2^ = 0.004]. However, there was a significant Validity × Orientation interaction [*F*(1,118) = 13.49, *p* < 0.001, η_p_^2^ = 0.103]. Further comparisons revealed significant cueing effects for upright bicycles [*t*(59) = 6.01, *p* < 0.001, *d* = 0.776, CI_95%_ = 0.03, 0.07], but not for inverted bicycles [*t*(59) = 0.31, *p* = 0.756, *d* = 0.038, CI_95%_ = − 0.02, 0.02].

#### Cars

120 participants (58 female, 60 male, 2 non-binary) with an age range of 18 to 60 years (*M*_age_ = 34.3, *SD*_age_ = 10.8) were recruited through Prolific. Trials where participants responded incorrectly (1.7%) were excluded from the analysis. All participants performed correctly on at least 60 of the 64 catch trials. RTs were subjected to ANOVA with Validity (valid, invalid) as a within-subjects factor and Orientation (upright, inverted) as a between-subjects factor. The analysis revealed a significant main effect of Validity [*F*(1,118) = 18.62, *p* < 0.001, η_p_^2^ = 0.136], whereby participants responded faster on valid trials, than on invalid trials. We observed no main effect of Orientation [*F*(1,118) = 0.11, *p* = 0.741, η_p_^2^ = 0.001] and no Validity × Orientation interaction [*F*(1,118) = 1.01, *p* = 0.317, η_p_^2^ = 0.008]. Further comparisons revealed significant cueing effects for upright [*t*(59) = 3.55, *p* < 0.001, *d* = 0.457, CI_95%_ = 0.03, 0.10] and inverted cars [*t*(59) = 2.50, *p* = 0.015, *d* = 0.320, CI_95%_ = 0.01, 0.07].

## General discussion

Previous work has established that faces, bodies, and objects cue observers’ visuospatial attention. For example, participants find target items faster when the correct location (on the left or right of the display) is cued by the directionality (facing-left vs. facing-right) of a task-irrelevant face^4^, body^5^, or object^26^. The present study sought a better understanding of these cueing effects by examining their sensitivity to cue orientation. Previous results suggest that the cueing effects produced by faces and bodies are sensitive to the orientation of the cue^4,5^. However, it is unclear whether sensitivity to orientation is a unique hallmark of social attention cueing, or whether this is a more general phenomenon.

Our first set of experiments focussed on the cueing effects produced by faces and bodies (both with and without the head visible). The results were remarkably consistent. In all experiments, we found that cueing effects induced by face- and body-direction are greatly reduced by orientation inversion, replicating previous effects^4,5^. While upright exemplars produced highly significant cueing effects, inverted faces and bodies did not (irrespective of head visibility).

Our second set of experiments focussed on the cueing effects produced by six common objects (power drills, desk lamps, desk fans, cameras, bicycles, and cars). When cueing stimuli were shown upright, all six object classes produced highly significant cueing effects, replicating previous findings^26^. When shown upside-down, however, the results were mixed. Some of the cueing effects (e.g., those induced by bicycles and cameras) behaved liked faces and bodies: they were greatly reduced by orientation inversion. However, other cueing effects (e.g., those induced by cars and power drills) were insensitive to orientation: upright and inverted exemplars produced significant cueing effects of comparable strength.

The results from these experiments defy a simple explanation whereby social and non-social cues behave in qualitatively different ways. However, the processing delay hypothesis^5^ offers a framework within which these findings may be understood. According to this view, a stimulus will only cue visuospatial attention when its directionality is inferred very quickly. Some cueing effects may be abolished or attenuated by stimulus inversion because exemplars are processed less efficiently when encountered upside-down^5^. Other cueing effects may be relatively insensitive to orientation inversion because observers can identify the directionality equally quickly when exemplars are encountered upright or upside-down.

All of the objects used in the study have a canonical front and back. Across the different stimulus classes, however, different types of visual feature signal that directionality. For some objects (e.g., cameras, bicycles), these diagnostic cues are quite subtle (e.g., the relative positioning of the camera lens or the bicycle handlebars). Indeed, many parts of these images hardly change in leftward and rightward facing exemplars (e.g., the wheels of a bicycle, or the stand of a camera). Orientation-specific perceptual expertise may be particularly important for the rapid location and interpretation of these subtle direction cues. For other types of object (e.g., power drills, cars), there are crude differences in stimulus outline that distinguish leftward and rightward facing exemplars. Perceptual expertise may be relatively unimportant for the rapid identification of these crude direction cues.

It would be interesting for future research to examine whether the speed with which participants make categorisation decisions (e.g., facing-left or facing-right?) about the directionality of object cues (e.g., power drills, desk fans, desk lamps, cameras, bicycles, cars), varies as a function of exemplar orientation (upright or inverted). If our interpretation is correct, it predicts that speeded categorisation decisions about exemplar directionality ought to be slower for inverted cameras and inverted bicycles, relative to upright cameras and upright bicycles. However, inversion should have little or no detrimental effect on speeded decisions about power drills and cars. It might also be interesting to examine the effects of stimulus inversion on the cueing effects seen with object experts. It is possible that lab-based training that augments expertise with object cues, may render the cueing effects produced by these stimuli more susceptible to inversion.

Much has been written about the ability of social attention cues^1–3^ and arrows^29,30^ to direct observers’ visuospatial attention. To date, however, the ability of common objects to direct visuospatial attention has been largely over-looked. Our findings confirm that several common objects direct observers’ visuospatial attention in an automatic (i.e., hard-to-inhibit) manner. Different objects may cue participants’ visuospatial attention for different reasons. For example, tools may cue attention in the direction implied by their likely use^31^, and vehicles may cue attention in their expected direction of travel^26^. Desk fans, desk lamps, and cameras may come to cue attention because their presence and positioning predict the location of items of interest in the visual environment^26^. We note, however, that several objects with a canonical front and back do not cue attention, including guns, shoes and chairs^26^.

There has been much interest in whether the attention cueing induced by faces and bodies is mediated by domain-specific or domain-general mechanisms^32–34^. It is clear from our results that sensitivity to orientation is not a unique hallmark of social attention cueing. Some non-social cueing effects, notably those produced by bicycles and cameras are also greatly reduced by inversion. We cannot exclude the possibility that orientation disrupts the cueing by social and non-social stimuli for different reasons. However, the fact that inversion disrupts attention cueing by faces and bodies can no-longer be cited as evidence that social cueing effects are ‘special’.

Despite the present results, it remains possible that the mechanisms that mediate social and non-social cueing effects do dissociate. There is some suggestion that the cueing effects produced by social attention cues, arrows, and objects, have different temporal characteristics; for example, the cueing effects seen for objects may manifest a little later^31^. Observers may also be better able to inhibit direction cueing by non-social cues (arrows) than by gaze^34^. Similarly, neuropsychological evidence suggests that the cueing effects produced by arrows and gaze-cues may dissociate^33^. Finally, gaze cueing effects appear to be moderated by social factors, including the relationship between the observer and the cueing face^35^. It is unclear whether analogous influences (e.g., effects of familiarity or ownership) are seen with the attention cueing induced by arrows and objects.

It is well-known that perceptual decisions about faces are harder and less accurate when target faces are shown upside-down^36–38^. While comparable effects have been found with body stimuli^39,40^, it remains unclear whether the visual processing of bodies is impaired by stimulus inversion, or whether the disruption observed is a by-product of face and head inversion^22^. Given this uncertainty, it is noteworthy that the directional cueing effects induced by our body stimuli were attenuated by orientation inversion even when the face and head were occluded by a grey oval. This result is indicative of a body (not head) inversion effect, and accords with the view that faces and bodies engage similar forms of visual processing^41–43^. One possibility is that bodies and faces both engage orientation-specific perceptual expertise that supports the rapid efficient processing of upright, but not inverted, exemplars^16,18^.

Having first confirmed that the cueing effects produced by faces and bodies are sensitive to orientation, we examined whether the cueing effects produced by objects are also reduced by inversion. While some cueing effects were attenuated by inversion (e.g., the effects produced by bicycles and cameras), several others were unaffected by this manipulation (e.g., the effects produced by power drills, desk fans, and cars). We speculate that (i) cueing effects depend on the rapid identification of stimulus directionality, and (ii) some cueing effects are sensitive to orientation because upright exemplars of those categories afford faster processing of directionality, than inverted exemplars. Contrary to the view that attenuation-by-inversion is a unique hallmark of social attention, our findings indicate that some non-social cueing effects also exhibit sensitivity to orientation.

## Data Availability

Data for all experiments can be accessed here: https://osf.io/sx7tv/.
